# COVID-19 Immunobiology: Lessons Learned, New Questions Arise

**DOI:** 10.3389/fimmu.2021.719023

**Published:** 2021-08-26

**Authors:** Aimilios Kaklamanos, Konstantinos Belogiannis, Panagiotis Skendros, Vassilis G. Gorgoulis, Panayiotis G. Vlachoyiannopoulos, Athanasios G. Tzioufas

**Affiliations:** ^1^Department of Pathophysiology, School of Medicine, National and Kapodistrian University of Athens, Athens, Greece; ^2^Institute for Autoimmune Systemic and Neurological Diseases, Athens, Greece; ^3^Molecular Carcinogenesis Group, Department of Histology and Embryology, School of Medicine, National and Kapodistrian University of Athens, Athens, Greece; ^4^First Department of Internal Medicine and Laboratory of Molecular Hematology, University Hospital of Alexandroupolis, Democritus University of Thrace, Alexandroupolis, Greece; ^5^Faculty Institute for Cancer Sciences, Manchester Academic Health Sciences Centre, University of Manchester, Manchester, United Kingdom; ^6^Basic Research Center, Biomedical Research Foundation of the Academy of Athens (BRFAA), Athens, Greece; ^7^Center for New Biotechnologies and Precision Medicine, School of Medicine, National and Kapodistrian University of Athens, Athens, Greece

**Keywords:** SARS-CoV-2, COVID-19, immune deregulation, immunothrombosis, senescence, endothelitis, autoimmunity

## Abstract

There is strong evidence that COVID-19 pathophysiology is mainly driven by a spatiotemporal immune deregulation. Both its phenotypic heterogeneity, spanning from asymptomatic to severe disease/death, and its associated mortality, are dictated by and linked to maladaptive innate and adaptive immune responses against SARS-CoV-2, the etiologic factor of the disease. Deregulated interferon and cytokine responses, with the contribution of immune and cellular stress-response mediators (like cellular senescence or uncontrolled inflammatory cell death), result in innate and adaptive immune system malfunction, endothelial activation and inflammation (endothelitis), as well as immunothrombosis (with enhanced platelet activation, NET production/release and complement hyper-activation). All these factors play key roles in the development of severe COVID-19. Interestingly, another consequence of this immune deregulation, is the production of autoantibodies and the subsequent development of autoimmune phenomena observed in some COVID-19 patients with severe disease. These new aspects of the disease that are now emerging (like autoimmunity and cellular senescence), could offer us new opportunities in the field of disease prevention and treatment. Simultaneously, lessons already learned from the immunobiology of COVID-19 could offer new insights, not only for this disease, but also for a variety of chronic inflammatory responses observed in autoimmune and (auto)inflammatory diseases.

## Introduction

Coronavirus Disease-2019 (COVID-19) has costed millions of lives worldwide. Since the beginning of the pandemic, the scientific community has joined forces to understand the pathophysiology of the disease, aiming to develop effective preventive and therapeutic measures. The development of many vaccines complementing social and personal protective measures (distancing, masks, hand washing etc.) coupled with pharmacologic treatments (heparin, dexamethasone, remdesivir and others), constitute our current armamentarium against Severe Acute Respiratory Syndrome Coronavirus-2 (SARS-CoV-2). Despite such a collective effort, we are still far from altering the disease’s clinical course, particularly the devastating course of many cases. In this review, we will summarise, in a step-by-step approach, the existing evidence regarding the key pathophysiological processes that take place from viral inhalation to disease establishment in all its forms, with emphasis on the immunopathology of the disease and the differences among the diverse clinical courses. Lessons learned from the immunopathology of COVID-19 hitherto, including the interplay between innate and adaptive immune responses, the immune-mediated thrombosis, panoptosis, autoimmunity and cellular senescence, could offer new insights, not only for the disease per se, but also for a variety of chronic inflammatory responses observed in autoimmune and (auto)inflammatory diseases.

## SARS-CoV-2: From inhalation to COVID-19

### Viral Effects on the Respiratory System

**Figure 1:**
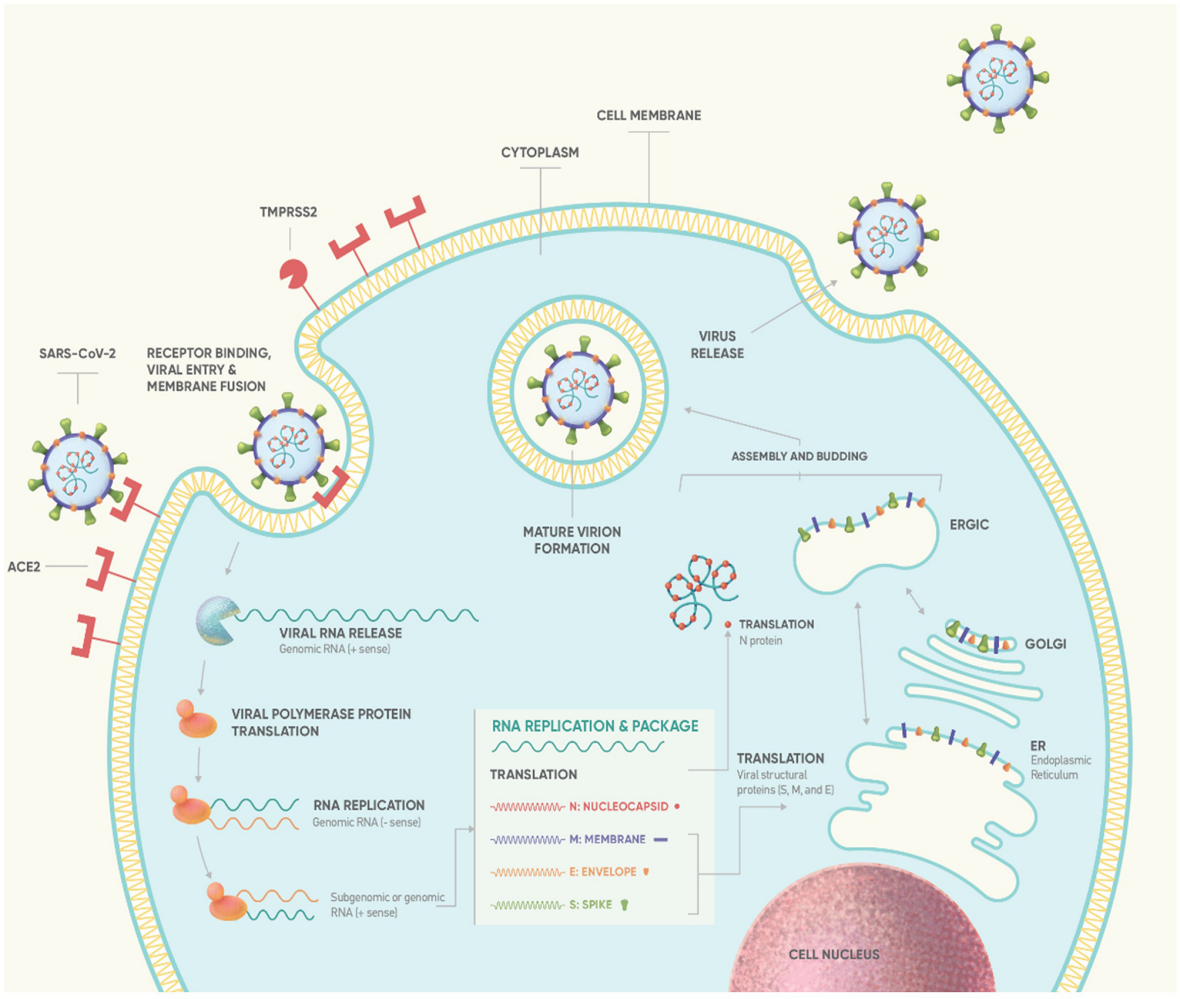
Life cycle of SARS-CoV-2 (13). Binding of S protein to ACE2 receptor induces the proteolytic cleavage of S protein by cell surface-associated transmembrane protease serine 2 (TMPRSS2) and cathepsin. S1-RBD is recognized by ACE2 (14), mediating virus fusion and entry into the target cell *via* endocytosis (13, 15). Subsequently, the virus releases its positive-sense RNA-strand into the cytoplasm which serves as template to produce full length negative-sense RNAs by a reaction catalyzed by RTC. Using ribosomes of infected cell, virus ORF1a and ORF1ab are translated producing the pp1a and pp1ab polyproteins. Replication of viral RNA and N protein is then followed, mediating the packaging of viral RNA. Simultaneously, genes encoding S, M and E protein are transcribed and translated in the ER and transported to Golgi complex to form ER–Golgi intermediate compartment (ERGIC). Virus assembly and budding is mediated through the interaction between the RNA-N complex and the ERGIC creating a mature virion. Finally, the newly formed virion is released from host cell *via* exocytosis (13, 15, 16). According to estimated data of viral dynamics during *in vitro* experiments, SARS-CoV-2 requires approximately 10min to enter susceptible cell lines and about 10hrs to replicate intracellularly (the so-called eclipse period) such that 10^3^ progeny virions are released from a single cell (‘burst’ size: ~10^3^) through continuous budding (17). ACE2, angiotensin-converting enzyme 2; ER, endoplasmic reticulum; RBD, receptor-binding domain; RTC, replicase-transcriptase complex.

**Figure 2:**
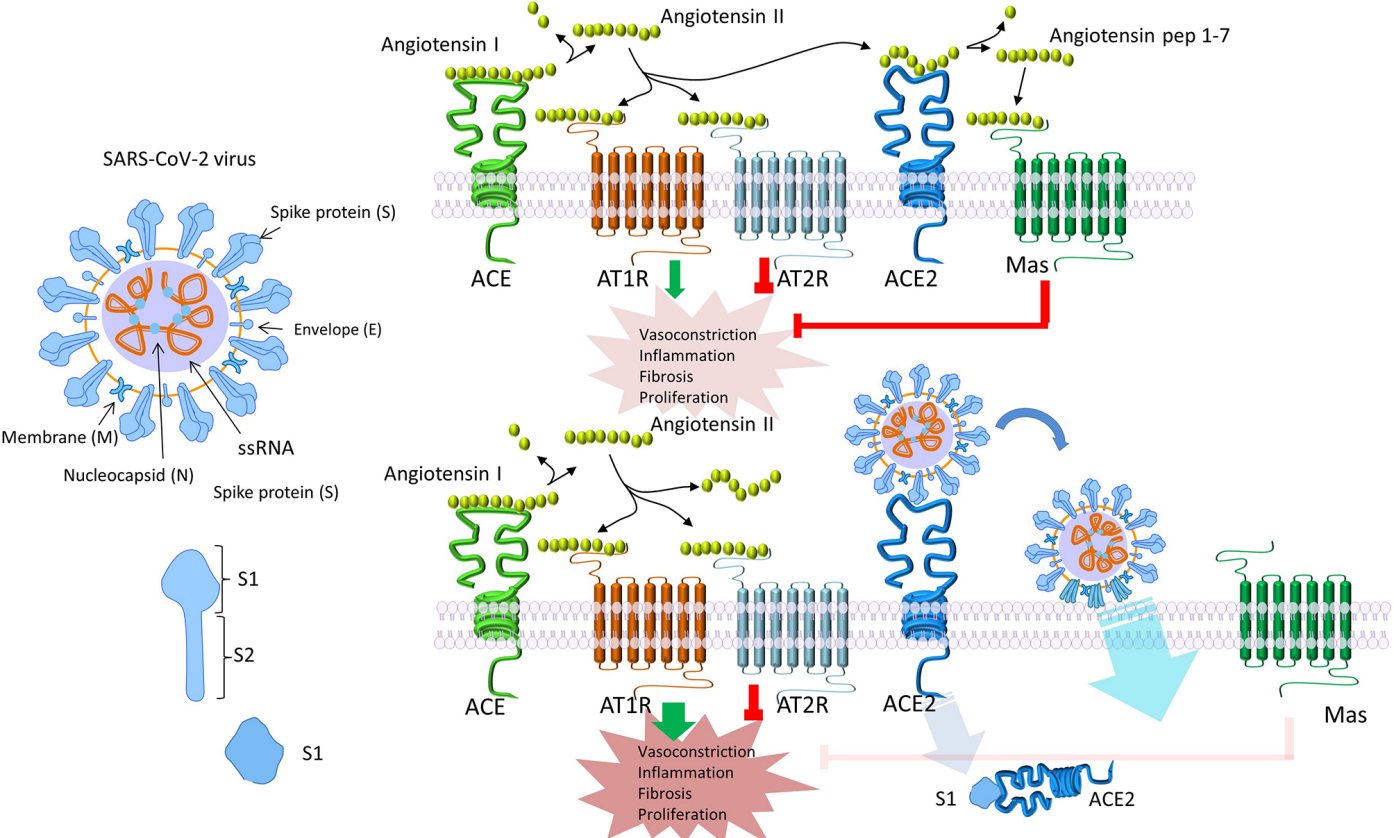
SARS-CoV-2 endocytosis alters angiotensin II signaling. Following endocytosis of SARS-CoV-2 together with its receptor ACE2, the number of available ACE2 molecules on the cell membrane is decreased (20). As a result, circulating angiotensin II is not degraded to angiotensin_1-7_. Angiotensin II signals mainly through AT1, while angiotensin_1-7_ through AT2 and Mas receptors. Signaling through AT1, increases vasoconstriction and interstitial fibrosis, enhances inflammation by macrophage activation and, eventually, the production of inflammatory cytokines, induces endothelial dysfunction, and increases pulmonary permeability leading to pulmonary edema and ARDS (23). Reduced expression of ACE2 can indirectly activate the kinin-kallikrein system, leading to increased vascular permeability (24). Simultaneously, downregulation of ACE2 has been connected to myocardial hypertrophy and dysfunction, obesity-associated hypertension and increased oxidative stress (20, 25, 26). Interestingly, in many conditions which serve as high risk factors for COVID-19, like increased age, male gender, obesity, diabetes mellitus, arterial hypertension, and heart insufficiency, ACE2 is downregulated or deficient (20, 27–29). The subsequent, already increased AT1 signaling, could, at least partially, explain the increased risk and the worse clinical progression of COVID-19 in these patients (20). To the contrary, signaling through AT2 or Mas receptors, has the opposite effects and therefore seems to confer a protection against angiotensin II–AT1 signaling (20, 30). ACE2, angiotensin-converting enzyme 2; AT, angiotensin II receptor.

SARS-CoV-2 is responsible for the so-called COVID-19, an airborne disease affecting initially the respiratory tract and eventually leading to a systemic disorder (1) (**Table 1**) (2–12). Through inhalation of air droplets and/or direct virus inoculation on mucosal surfaces, this novel viral strain can infect specific cells, of both the upper and lower respiratory tract, particularly those expressing high numbers of the Angiotensin Converting Enzyme-2 (ACE-2) receptor (**Figure 1**) (14–18). The ciliated epithelial cells appear particularly sensitive to this virus. Moving along the airways towards the lower parts of the respiratory tract, ACE-2 expression levels drop steadily, resulting in lower infection rates of type II pneumocytes (19). Ciliated epithelial cells and type II pneumocytes along with the resident lung macrophages -both alveolar and interstitial types- and vascular endothelial cells, are the main cellular targets for SARS-CoV-2 in the respiratory system (20, 21). Beyond the respiratory system, ACE2 has a ubiquitous expression in other human tissues and organs including the heart, kidney, intestine, vessels, liver, central nervous system, eyes etc. (22), and therefore, SARS-CoV-2 can potentially invade many different cells/tissues. Following the endocytosis of SARS-CoV-2 coupled with its receptor ACE2, the number of available molecules of ACE2 on cell membrane is reduced (20). As a result, the circulating angiotensin II is not degraded to angiotensin_1-7_ and the consequences of this phenomenon are depicted in **Figure 2** (20, 23–25, 27–30).

**Table 1:** COVID-19 is a systemic disease affecting many different organs/systems.

Systemic symptoms (2)	Mild: fever, malaise, fatigue, cough, dyspnea, chest pain, nasal congestion, and sore throat Severe: pneumonia, pulmonary edema, acute respiratory failure, ARDS, sepsis, multiorgan failure, shock, and death
cardiovascular system (3–5)	myocardial injury, myocarditis, acute coronary syndrome, heart failure, arrhythmia, sudden cardiac death, or thrombosis with subsequent thromboembolic events like ischemia, deep venous thrombosis and pulmonary embolism
gastrointestinal system (6, 7)	loss of appetite, diarrhea, nausea, vomiting and abdominal pain, elevated liver enzymes (bilirubin, AST, ALT), while the virus can be found in stool samples
kidneys (8)	acute kidney injury
nervous system (9)	headache, dizziness, anosmia, ageusia, encephalopathy, encephalitis, ischemic stroke, neuroinflammation or rarely immune-mediated complications such as ADEM, acute necrotizing hemorrhagic encephalopathy or Guillain-Barre syndrome
skin (10)	maculopapular, morbilliform, urticarial, vesicular, chilblain-like, petechiae/purpura and livedoid, or maculo-papular, or erythema multiforme-like, or diffuse erythroderma rash associated with a hyperinflammatory Kawasaki-like syndrome in children
muscular system (11)	myopathy
hematologic parameters (12)	elevated CRP - LDH - ferritin - CK levels, decreased albumin and elevated levels of various inflammatory cytokines like TNFα and IL-6

ADEM, acute disseminated encephalomyelitis; ARDS, cute respiratory distress syndrome; ALT, alanine aminotransferase; AST, aspartic aminotransferase; CK, creatine kinase; CRP, C-reactive protein; LDH, lactate dehydrogenase.

Mild and severe refer to the severity of systemic COVID-19 symptoms. So it is “Mild symptoms” and “severe symptoms”.

In parallel, viral infection of host cells activates both the innate and adaptive arms of the immune system, mounting an immune response against SARS-CoV-2. Robust aberrant cellular alterations, including cellular senescence (31), apoptosis, with emphasis in the inflammatory subtypes thereof [pyroptosis (32), necroptosis (33), PANoptosis (34)] and NETosis are implicated in the disease course and severity.

### Innate Immune Response Against SARS-CoV-2

**Figure 3:**
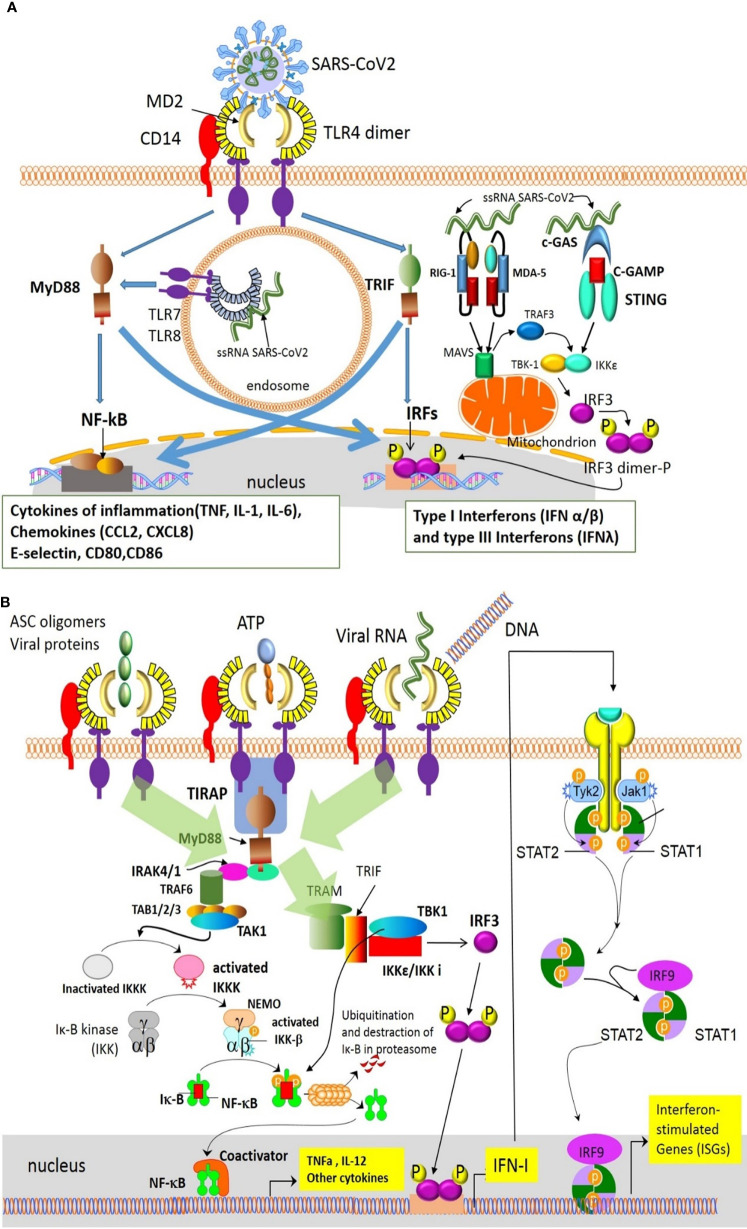
Innate immune detection of SARS-CoV-2 results in the production of interferons, cytokines and chemokines (35). Innate immune cells detect SARS-CoV-2 *via* different mechanisms/pathways. **(A)** Viral RNA activates the MyD88–NF-kB and IRF pathways inducing production of inflammatory cytokines and type I interferons. Additionally, the cGAS–STING and the MDA-5/RIG-I pathways, also sense the cytoplasmic viral RNA or viral replicative intermediates, and lead to the production of type I and III interferons (IFNa/β and IFNλ). In turn, these interferons, acting in an autocrine/paracrine way *via* the IFNAR1/2 or IFNAR2/IL10R pathways, induce the expression of ISGs. **(B)** PRRs like TLR4 also recognize PAMPs-DAMPs released by damaged cells and induce the production of inflammatory cytokines through the NF-kB pathway (36). ASC, apoptosis-associated speck-like protein containing a CARD; cGAMP, cyclic GMP-AMP; cGAS, cyclic GMP-AMP Synthase; DAMPs, damage-associated molecular patterns; IFN, interferon; IFNAR, interferon receptor; IKK, inhibitor of NFkB; IRAK , IL-1R associated kinase; IL10R,interleukin-10 receptor; IRF, interferon regulatory factor; ISGs, interferon-stimulated genes; Jak, Janus kinase; MAVS, mitochondrial antiviral-signaling protein; MD2, myeloid differentiation factor 2; MDA-5, melanoma differentiation-associated gene 5; MyD88, Myeloid differentiation primary response 88; NEMO, NFkB essential modulator; NF-kB, nuclear factor kappa B; PAMPs, pathogen-associated molecular patterns; PRRs, Pattern Recognition Receptors; RIG-I, retinoid inducible gene I; STAT, Signal transducer and activator of transcription; STING, Stimulator of Interferon Genes; TAB, Mitogen-activated protein kinase kinase binding protein; TAK1, TGFβ-activated kinase 1; TBK-1, TANK-binding kinase 1; TIRAP, Toll-interleukin 1 receptor (TIR) domain containing adaptor protein; TLR4, Toll-like receptor 4; TRAM, Translocating chain-associated membrane protein; TRAF, TNF receptor-associated factor; TRIF, TIR-domain-containing adapter-inducing interferon-β; Tyk, Tyrosine kinase.

Innate Immunity, which is the first line of defence in response to infection, is initiated by Pattern Recognition Receptors (PRRs)-mediated identification of Damage-Associated Molecular Patterns (DAMPs) and Pathogen-Associated Molecular Patterns (PAMPs). The former are released by the infected cell and the latter are virus-associated molecules, such as ssRNA, acting as danger signals, which eventually activate the immune system. More specifically, as depicted in **Figure 3** (35, 36), Toll-like receptors (TLRs) bind their ligands, and subsequently activate cytosolic RLRs and NLRs (37–39), leading to the production of interferons, cytokines and chemokines.

The earliest innate antiviral events, occurring within hours post infection, are Type I and Type III Interferon (IFN)-responses, that act as barriers to viral replication and dissemination. Type I interferons can directly inhibit the viral replication and enhance both innate and adaptive immunity, *via* various mechanisms, including: 1) MX1–GTPase polymerization, 2) IFN-inducible dsRNA-dependent protein kinase R (PKR), 3) 2ʹ-5ʹ- oligoadenylatesynthetase (OAS), 4) IFN-induced transmembrane protein (IFITMs), 5) APOBEC1 and the TRIM family of molecules (40). The main cellular source of type I IFNs in response to SARS-CoV-2, appears to be the plasmacytoid Dentritic Cells (pDCs), activated *via* the TLR7, as is the case for other coronaviruses (41).

**Figure 4:**
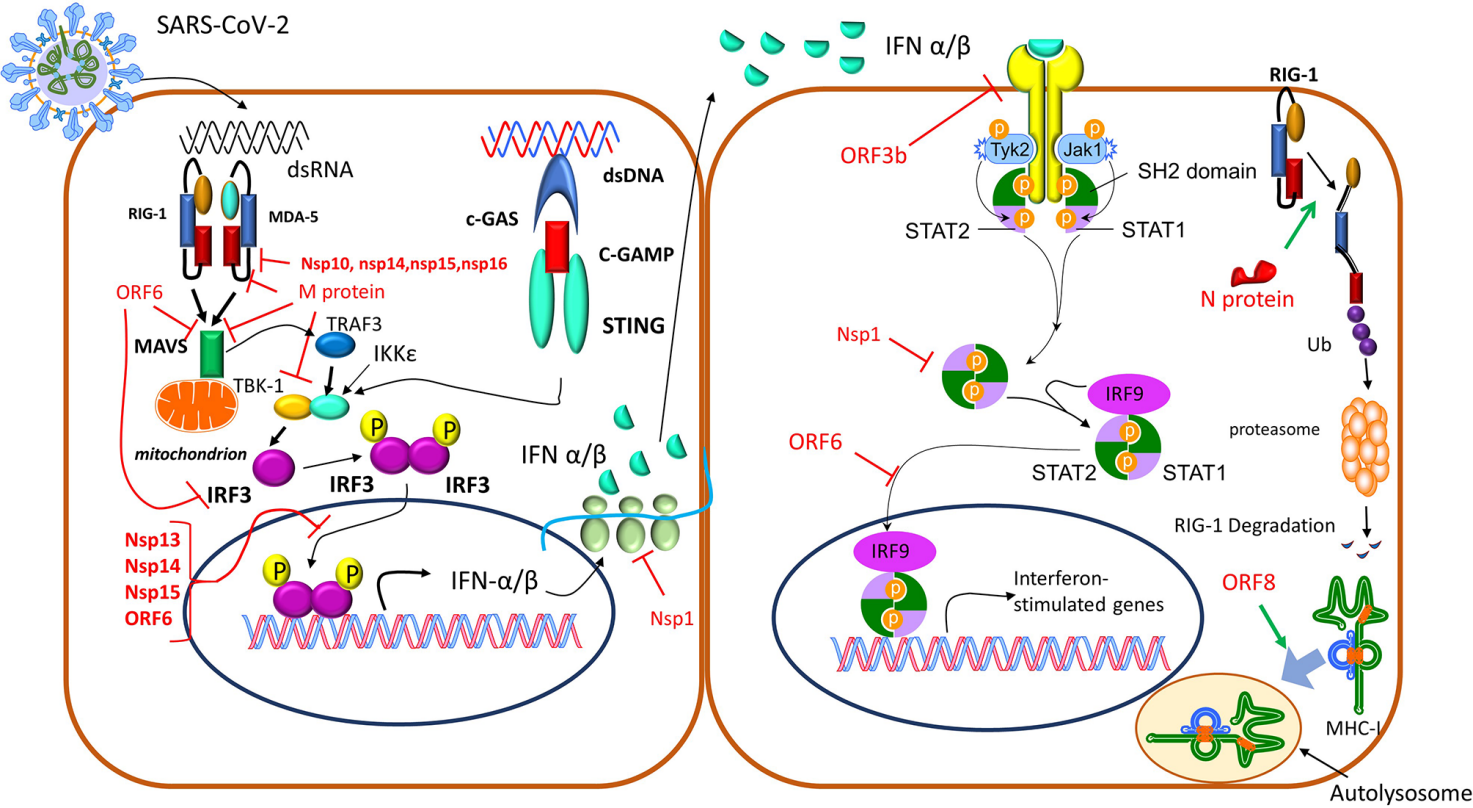
Corona viruses exploit different mechanisms to avoid immune detection (35, 41, 46). 1) The N protein-targeted ubiquitination drives RIG-I for proteasomal degradation, depleting the cell of a critical PRR. 2) Furthermore, the IFN pathway is inhibited by SARS-CoV-2’ proteins through various mechanisms: a) ORF3b, highly expressed in the acute stage of the infection, downregulates the expression of IFNAR, and eventually IFN I responses. b) Nsp13, Nsp14, Nsp15, and ORF6 suppress IRF3 nuclear translocation, c) Nsp1, blocks mRNA translation and also decreases STAT phosphorylation, d) M protein as well as nsp10, nsp13, nsp14, nsp15, can inhibit the production of type I and III IFNs through interaction with the RIG-I/MDA-5-MAVS signaling pathway, e) ORF8 can directly bind to MHC I molecules at the endoplasmic reticulum and redirect them to autolysosomes for degradation, which results in its inefficient elimination by cytotoxic lymphocytes. IFN, interferon; IFNAR, interferon a receptor; IRF3, interferon regulatory factor 3; MAVS, mitochondrial antiviral-signaling protein; MDA-5, melanoma differentiation-associated gene 5; MHC, major histocompatibility complex; PRR, Pattern Recognition Receptor; RIG-I, retinoid inducible gene I; STAT, Signal transducer and activator of transcription.

These pathways have attracted the attention of current research since coronaviruses (CoVs) possess an intrinsic capacity to interfere with various IFN pathway-related molecules. Indeed, the implementation of specific structural and non-structural proteins has revealed that SARS-CoV-2 induces diminished or delayed Type I Interferon responses (42–44), thus generating, early in the disease course, a replication permissive microenvironment. This phenomenon probably explains the high viral loads upon the onset of disease symptoms seen in COVID-19 patients (41), the unusually long viral incubation time and the high rates of asymptomatic or pauci-symptomatic patients (44). The mechanisms utilized by the coronaviruses to evade the initial immune recognition and response *via* IFNs can be divided into three categories: (1) avoidance of the recognition by the PRRs, (2) suppression of the transcription of interferons, and (3) the suppression of IFN signaling by the viral proteins through inhibition of IFNAR signaling or autoantibodies against IFN pathway elements that neutralize IFN actions. The precise mechanisms used by SARS-CoV-2 are yet unknown, but their majority is expected to share the same basis among different coronaviruses (45) as shown in **Figure 4** (35, 41, 46). In addition to these mechanisms, SARS-CoV-2 uses its own capping machinery (nsp10, nsp13, nsp16), to generate 2-o-methyltransferase caps. These RNA caps are indistinguishable from cellular mRNA caps, therefore, the RNA detection and degradation by the type I IFN- inducible proteins, MDA-5 and IFIT, are circumvented. Furthermore, SARS-CoV-2 uses replicase–transcriptase complexes that protect the virus during the maturation process. Moreover, corona viruses use glycans and/or other post-translational modifications to mask the immunogenic viral protein epitopes (35). SARS-CoV-2 can also infect antigen-presenting cells (APCs), inducing epigenetic changes that cause an HLA-II downregulation, as described in both monocytes/macrophages, and B cells (47), probably *via* an IL-6-dependent pathway (48). Furthermore, the blockade of interferon signaling limits the Interferon-Stimulated Genes (ISGs) expression in APCs, including the cytokine-induced MHC-II expression. Moreover, SARS-CoV-2 protein Nsp5 interacts with the epigenetic regulator Histone Deacetylase 2 (HDAC2), which controls the MHC-II expression and cytokine production (49, 50). Finally, ORF8 of SARS-CoV-2 can directly bind to MHC-I molecules in the ER, redirecting them to autolysosomes for degradation and resulting in the inefficient elimination of the virus by Cytotoxic Lymphocytes (CTLs) (46).

Normally, Type I and III IFN responses activate and augment the function of immune system tissue resident cells including dendritic cells, alveolar and interstitial tissue resident macrophages and Natural Killer cells (NK-cells). For example, monocytes/macrophages phagocytose the infected cells and induce type I IFN responses and pro-inflammatory molecules. NK cells recognize peptides expressed on the surface of infected cells and destroy them *via* direct cytotoxicity, through perforins and granzyme B. All immune cells release cytokines and chemokines, such as among others, TNF-a, IL1β, IL6 and IL8, that amplify and propagate the immune response by recruiting more cells in the following time order: Neutrophils are recruited first (within 12-24 hours post-infection, usually peaking at day 2-3), followed by pDCs (41). Monocytes, T- and B-cells appear later, from day 4-5 onwards, thus switching the immune response to its adaptive arm. Adequate adaptive responses require approximately 6-10 days of priming, reflecting the physiological lag period (post innate pathogen sensing) that is needed for the generation of pathogenspecific T- and B- cell clonal selection (51).

In parallel, glycans attached on viral proteins that are expressed on the infected cell surface can activate the lectin complement pathway after binding on the Mannose-Binding Protein (MBP). Complement activation leads to further inflammatory cell activation and the generation of Membrane Attack Complex (C5b-C9) on the surface of alveolar and epithelial cells, resulting in their destruction (52).

### Adaptive Immune Response Against SARS-CoV-2

A week after the onset of COVID-19 symptoms, if the above described mechanisms operate uninterrupted, adaptive immunity, - mediated by CD4^+^ T-cells, CD8^+^ cytotoxic T-cells and antibodies -, is activated (53). Glycans expressed on SARS-CoV-2 are recognized by DC-SIGN and other lectins, facilitating the viral uptake by DCs and pDCs, which in turn present viral antigens to CD4^+^ T-helper cells. This results in a Th1 response with enhanced IFN-γ, IL-2 and TNFα production/secretion (53, 54). CD8^+^ T-cell responses specific to SARS-CoV-2 also produce IFN-γ, IL-2 and TNFα, resulting in direct cytotoxicity against infected cells (55). Notably, memory T-cells have also been detected in individuals that do not produce antibodies against SARS-CoV-2, which suggests that cellular immunity operates together and/or independently of humoral immunity (55). Lymphocytes, and in particular CD4^+^ T-cells, are the main regulators of the adaptive immune response against the invader, and their chemotaxis to the lungs might be partly responsible for the lymphopenia observed in the peripheral blood of many COVID-19 patients, a hallmark of COVID-19 (56, 57).

Follicular T-helper cells, through the formation of germinal centers and the provision of B-cells with all the necessary co-stimulatory molecules (such as CD40L and cytokines like IL-21), drive the humoral immunity towards SARS-CoV-2 surface glycoproteins, mainly glycoprotein S and nucleocapsid protein (58, 59). Antibodies against the spike glycoprotein and especially its Receptor Binding Domain possess a neutralizing effect against SARS-CoV-2, preventing the binding of the virus to ACE2 (60). Usually the first antibodies produced are of the IgA class, during the first week of infection, followed by IgM (in 10-12 days) and after the first 3 weeks by IgG (61). Interestingly, approximately 10% of infected COVID-19 patients have no detectable antibodies even after the 4^th^ week post symptom onset, leading to the hypothesis that antibody production is not required for SARS-CoV-2 resolution in these so-called “non-responder” patients. This paradox is apparently not the case, and a possible mechanistic explanation in a subset of these patients could be the masking of antibodies due to prolonged antigenemia/viremia (62), or some – until now not described in COVID-19 – antiidiotypic response. Although the clinical significance of the initial titers of the produced antibodies and the duration of humoral immune response are still under investigation (63, 64), it is widely accepted that irrespective of disease severity, both T- and B-cell function are critical for disease termination (51, 65).

## COVID-19 Immunobiology

### From Normal to Aberrant Host Immune Response

In physiology, the immune response is well orchestrated in time and space (spatiotemporally), in a way that its ultimate actions - resolution of damage, repair, and healing - are accomplished. Prerequisite for the latter is the counterbalance of pro-inflammatory actions with equal and opposite anti-inflammatory actions. For reasons still under investigation, this is not the case for a significant number of COVID-19 patients who experience the severe or critical form of the disease. According to latest research findings, however, spatiotemporally dys-synchronised and deregulated immune responses -starting early at the level of Type I and III IFN responses- appear to support the immunopathogenesis of COVID-19, tipping the balance towards an excessive, uncontrolled viral replication and the subsequent robust release of cytokines with detrimental effects on the human tissues. A good example are patients with either inborn errors in type I interferon signaling, in the genes coding for TLR3, UNC93B1, TICAM1, TBK1, IRF3, IRF7, IFNAR1, IFNAR2 (66) or preexisting neutralizing autoantibodies against type I IFNs (67), who are at higher risk for life-threatening COVID-19 infection. Genetic predisposition is also regarded as a crucial element to explain the transition from normal to aberrant host immune responses in different COVID-19 patients. Numerous studies have identified such genetic susceptibility loci, like apolipoprotein E and the ABO blood group genes, but the importance of these findings has not been understood to its entirety hitherto (68).

Comparative studies between mild and severe disease are a prerequisite for understanding essential differences governing the phenotypic switch between different COVID-19 disease states and for deciphering its pathophysiology. Recent reports have shown, that the transition from mild to moderate and ultimately to severe disease phenotype is coupled with metabolic, as well as innate and adaptive immune alterations. Multiomic analysis of mild, moderate, and severe cases has revealed a pivotal change occurring between mild and moderate disease states, rendering the therapeutic interventions very critical at the moderate stage. This change is characterised by the emergence of unusual cell types, including preferentially exhausted CD4^+^ T-cells, cytotoxic CD4^+^ T-cells, proliferative exhausted CD8^+^ T-cells (loss of CD8^+^ T-cell poly-functionality), S100^High^/HLA-DR^low^ monocytes and depletion of non-classical monocytes. This disturbance of cell populations is accompanied by metabolic depletion in lipids, amino acids and xenobiotics (69). Similarly, marked deregulation in lipid transport has also been shown by RNA-seq and high-resolution mass spectrometry, associated with abnormal complement activation, and neutrophil degranulation in severe cases (70). In line with that, two large multi-omic studies have demonstrated that in such critical cases, an abnormal myeloid cell compartment -associated with features of acute myelopoiesis with immature, dysfunctional neutrophils and monocytes- was observed (71, 72). Interestingly, both cell populations were reprogrammed to an anti-inflammatory or suppressive phenotype (71), which could reflect either a counter-regulatory attempt of the body to terminate the excessive inflammation or a futile attempt to eliminate the offending stimulus that persists. To this end, information around the timing of monocyte trafficking has also been studied with surge of CD169^+^ and re-appearance of CD16^+^ monocytic cells to be linked with elevated CCL2 and CCL3 levels seen in severe rather than mild disease (73). In support to the aforementioned, a recent transcriptomic analysis in the lungs of COVID-19 cases has revealed relative absence of cytotoxic cell signatures, thus creating a permissive setting for enhanced myeloid-lineage cell involvement in the destructive sequelae accompanying the disease process (74). As opposed to mild cases, CD8^+^ cytotoxic T-cells displayed impaired exhaustion features with enhanced cytotoxicity and inflammation and a robust CD8^+^ T cell memory with unclear pathogenetic role to date (75). Finally, the timing of IgG humoral immune response generation and its quality, dependent on both Fab-associated (epitope repertoire diversity, antibody affinity maturation, neutralizing potential) and Fc-associated functions (antibody isotype class switching along with FcR affinity binding and associated Fc phagocytic functions), were all critical factors that dictated disease progression and/or resolution (76, 77).

### Deregulated Interferon Responses

The first phenomenon observed in the aberrant host immune response against SARS-CoV-2 is an impaired induction of both type I and type III (IFN-λ) IFNs. Type I IFN levels tend to decline rapidly, whereas that of IFN-λ persist longer. Despite the limited ability of COVID-19 patients to produce IFNs, especially at the initial stage of the disease, they robustly express pro-inflammatory cytokines that can be maintained in the serum at high concentrations for a prolonged period of time. Delayed but persistent type I IFN responses may drive the excessive lung infiltration by monocytes/macrophages and neutrophils, as it has been shown for other CoVs (78). These cells can, in turn, produce high amounts of pro-inflammatory cytokines like TNFα, IL-6, IL-1β, as well as chemokines, that amplify the recruitment of immune cells on site and propagate the inflammatory response, thus contributing to cytokine deregulation (79). Findings from different patients suggest that some aspects of the disease, like the myopathy and the Pernio-like lesions (or COVID toes), may result from a type I interferonopathy. It is appreciated now that an initial robust type I IFN response can result in faster and better clearance of SARS-CoV-2, while a muted response, leads to viral persistence and severe disease (11, 80). In support of the above, patients with COVID-19 treated early with IFN-α2b exhibit reduced in-hospital mortality, whereas late application of therapy leads to increased mortality and delayed recovery. This observation highlights the crucial importance regarding the timing of IFN production in COVID-19 (44, 81).

### Cytokine Deregulation

The diminished interferon response leads to an ineffective viral clearance and a deregulated activation of the adaptive immunity, which in turn results in enhanced activation of the innate immunity as a compensatory mechanism. This condition further results in an aggressive uncontrolled inflammatory response and the release of a large amount of pro-inflammatory cytokines and chemokines which cause damage in different organs/systems. Circulating IL-6, IL-18, IFN-γ, IL-15, TNFα, IL-1α, IL-1β, IL-2, G-CSF, IP-10 (or CXCL10), MCP1 (or CCL2), MCP3 (CCL7) and MIP-1α (CCL3) are over-expressed in patients with moderate or severe COVID-19 (82). Studies have suggested a direct correlation between cytokine levels and lung injury, multiple organ failure, and an unfavorable prognosis. So far, no specific cytokine or cell has been identified as the driver of this process (83). This state has been referred, by some researchers, as “cytokine storm”. However, this term has been queried because the hyper-inflammatory phenotype seen in severe COVID-19 is different from other hyper-inflammatory conditions, since the cytokine levels are not particularly exacerbated (84).

Different inherent or acquired conditions including gender differences (female *vs* male) (85), inflammaging and other pre-existing conditions such as baseline immunosuppression, immune exhaustion and immunosenescence are additional factors which could affect this delicate balance (56, 86, 87). In elderly patients, inflammaging – a chronic low-grade sterile inflammation characterized by high baseline serum levels of CRP and cytokines like IL-6 and IL-8 (88)– along with the inflammation induced by SARS-CoV-2, can drive the expression of specific transmembrane molecules. Such molecules like the Major histocompatibility complex class I chain-related protein A (MICA) and B (MICB) are also expressed by senescent cells normally found on the respiratory epithelium. As a result, highly-differentiated senescent T-cells with an NK-like phenotype that express NK-receptors (NKG2D) are attrackted leading to the subsequent destruction of the epithelial cells (89).

Although a clear distinction between factors that drive the aberrant cytokine expression, and factors that are the result of this deregulation is in the majority of cases difficult to be pointed out, we will try to address these two parameters separately.

## Drivers of Cytokine Deregulation

### Immune-Mediators

As presented earlier in this review, the cytokine over-expression is a result of a deregulated innate and adaptive immune response. In severe COVID-19 macrophages seem to play a central role through the excessive production of pro-inflammatory cytokines and chemokines (90). Macrophages from patients with severe COVID-19 exhibit lower expression of apolipoproteins which correlates with a pro-inflammatory phenotype (91). Resident pro-fibrotic (SPP1^high^) and inflammatory activated (FABP4^+^) macrophage populations are dominant in the lungs of patients with severe COVID-19 (92). In addition, activated monocytes/macrophages can express tissue factor (TF) and promote thrombosis (93). Moreover, peripheral blood neutrophils of severe COVID-19 patients show increased numbers and marked hyper-responsiveness, as attested by the enhanced degranulation, neutrophil extracellular traps (NETs) release and pro-inflammatory cytokine production (94). An increased neutrophils/lymphocytes ratio has been associated with a higher morbidity and mortality of COVID-19 (95). Mast cell activation and the release of various vasoconstrictive or inflammatory mediators, like histamine, tryptase, chymase, IL-1β, IL-6, TNF-α, CCL2, GM-CSF and CXCL10, contribute to local inflammation, tissue destruction, and cytokine imbalance (96). Additionally, the decreased numbers of CD4^+^ T-cells observed in severe COVID-19 can contribute to hyper-inflammation *via* the impaired regulatory mechanisms of the inflammatory process or *via* impaired adaptive T- and B- cell responses (23, 97). Finally, increased IL-17 production by Th17 cells in COVID-19 patients has broad pro-inflammatory effects through the upregulation of pro-inflammatory cytokines like G-CSF, IL-1β, IL-6, TNFα, as well as chemokines like MIP2A, IL-8, IP10, MIP3A, and matrix metalloproteinases (98).

### Cellular Stress-Response Mediators

#### Cellular Senescence

Cellular senescence is a stress response against a plethora of cellular insults, including effects of infectious agents through mechanisms of DNA damage, cell fusion and ER stress (31). It is characterized by 4 interconnected hallmarks of: cell cycle arrest, macromolecular damage, altered metabolism and pro-inflammatory features, called the senescence-associated secretory phenotype (SASP) (99). Such a response is activated upon accumulation of sub-lethal cellular damage that cannot be resolved by the physiological repair mechanisms and acts as fail-safe to prevent propagation of damage – viral dissemination, in the case of viral illnesses (100)- in both cellular and tissue level (101). Thus, its homeostatic role is widely accepted in physiology and pathophysiology, with its role in infectious diseases being still ill-defined (102). In some instances, cellular senescence is beneficial to the host since it obstructs viral replication and damage, while in others, it appears to facilitate viral-induced pathology (102, 103). Such a binary mode of action characterizes many facets of cellular senescence. It is likely that within this context, the fitness of the host (102), the timing of initiation of cellular senescence program and its duration, all together play key roles in the outcome of viral diseases like COVID-19. Within this context, we have recently showed that SARS-CoV-2 triggers senescence with various clinical and epidemiological implications, including quasispecies generation (31).

The above findings are of high clinical translational potential, as drugs specifically targeting the elimination of senescent cells, could alter disease clinical course once established, or even be used prophylactically prior to viral exposure. Nonetheless, large clinical trials are necessary for the establishment of the type and timing of such treatment strategies.

### Uncontrolled Inflammatory Cell Death – PANoptosis

**Figure 5:**
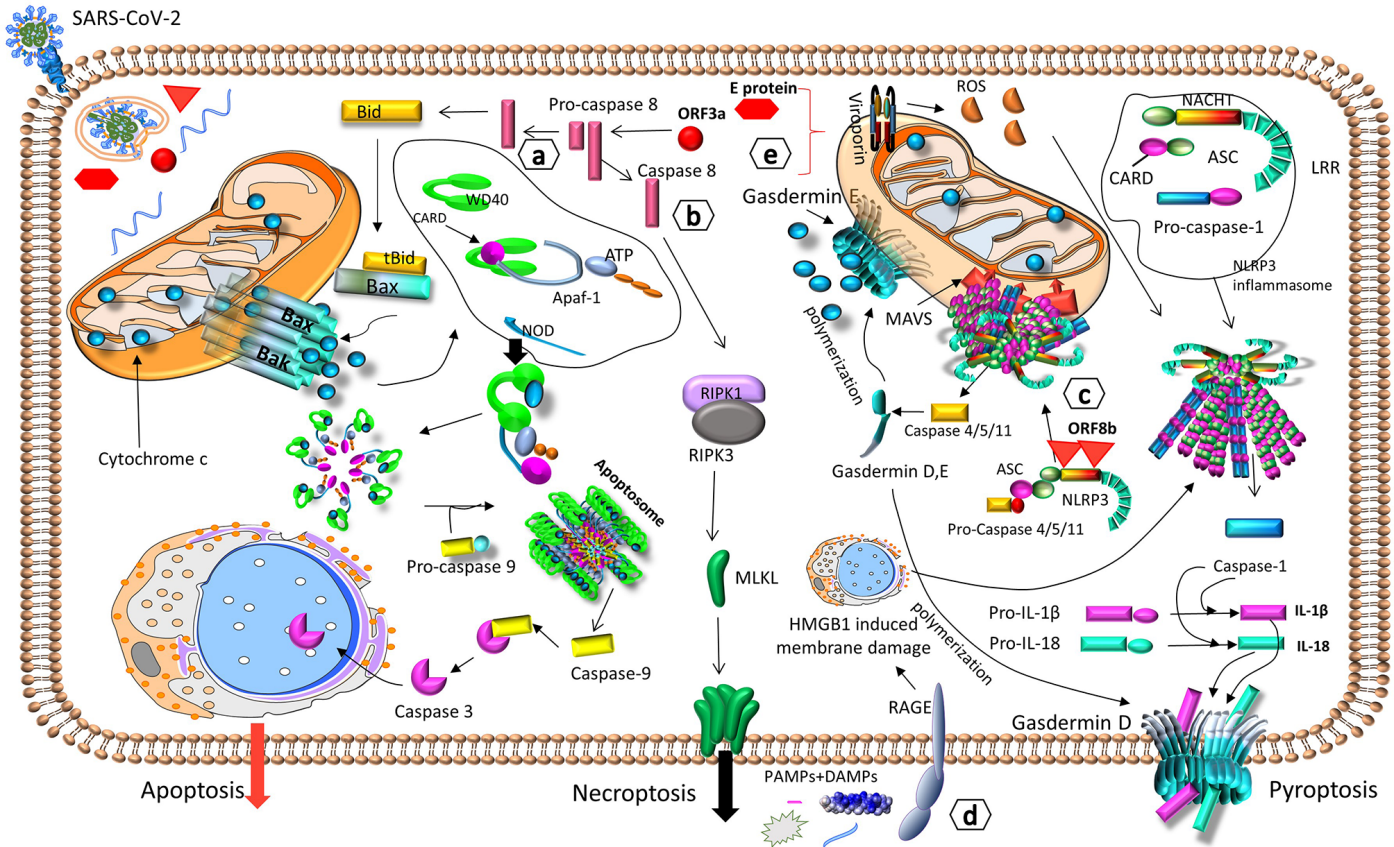
SARS-CoV-2 can trigger different types of cell death. SARS-CoV-2 induces cell death and destruction of target cells *via* a) apoptosis, through caspase 8-BID/tBID-cytochrome c-caspase 9 (104), b) necroptosis, through caspase 8-RIPK1-RIPK3-MLKL (33), c) pyroptosis, through NLRP3-mediated inflammasome activation and gasdermin D - caspases-1, -4, -5, -11 (105). All modes of cellular death result in the destruction of infected cells and the release of PAMPs, DAMPs, IL-1β and IL-18, which activate the innate immune response (33, 104, 105), d) PAMPs and DAMPs from damaged cells, can also be detected by the receptor of RAGE, which triggers HMGB1-induced membrane damage, NLRP3-mediated inflammasome activation, and pyroptosis (106). e) During intracellular viral replication, the viral proteins ORF3a and E, form viroporins, that augment ROS production, NLRP3-mediated inflammasome activation, and pyroptosis (107). Apaf-1, apoptotic protease activating factor 1; ASC, apoptosis-associated speck-like protein containing a CARD; ATP, adenosine triphosphate; Bac, Bcl-2 homologous antagonist/killer; Bax, Bcl-2-associated X protein; BID/tBID, BH3-interacting domain death agonist/truncated BID; CARD, caspase activation and recruitment domain; DAMPs, damage-associated molecular patterns; HMGB1, high mobility group box 1; IL, interleukin; LRR, leucine-rich repeat; MAVS, mitochondrial antiviral-signaling protein; MLKL, Mixed lineage kinase domain like; NACHT, NACHT domain; NLRP3, NOD-Like Receptor family pyrin domain containing 3; NOD, Nucleotide-binding Oligomerization Domain; PAMPs, pathogen-associated molecular patterns; RAGE, receptor of advanced glycation end products; RIPK, Receptor-interacting serine/threonine-protein kinase; ROS, reactive oxygen species; WD40, WD40 domain.

SARS-CoV-2 can trigger different types of cell death (**Figure 5**) (33, 104–107). Uncontrolled inflammasome activation and excessive pyroptosis, which is suggested to be the main “death mode” observed in COVID-19, can result in an hyper-inflammatory condition leading to severe disease, particularly in elderly patients (105). NLRP3, the major component of inflammasome, is already activated in older people as well as different pathological states that also serve as risk factors for severe COVID-19, including obesity, hypertension, cardiovascular disease, COPD and diabetes mellitus (108). Given that the threshold of inflammasome activation is very low in such patients, SARS-CoV-2 infection offers a significant additive effect predisposing them to uncontrolled inflammasome activation and subsequent hyper-inflammatory response (109). Interestingly, quite recently, a novel type of programmed inflammatory cell death called PANoptosis, has been described in murine models of COVID-19 exhibiting hypercytokinemia, similar to that described in severe COVID-19 in humans. The term PANoptosis is used to describe the simultaneous activation of Pyroptosis, Apoptosis and Necroptosis in the same cell, leading to its inflammatory death (34). More specifically, increased TNFα/IFN-γ signaling has been shown to activate the STAT1/IRF1/iNOs/NO pathway, which in turn leads to the formation of a cytoplasmic multimeric protein complex called PANoptosome. PANoptosome then acts as a molecular scaffold for the assembly and activation of effector molecules like RIPK1/RIPK3/FADD/caspase 8, subsequently followed by the contemporaneous activation of pyroptosis *via* GSDME, apoptosis *via* Caspase 3/7 and necroptosis *via* MLKL. This inflammatory cell death further enhances the inflammatory state by the release of PAMPs, DAMPs and other proinflammatory molecules (34).

## Consequences of Cytokine Deregulation

**Figure 6:**
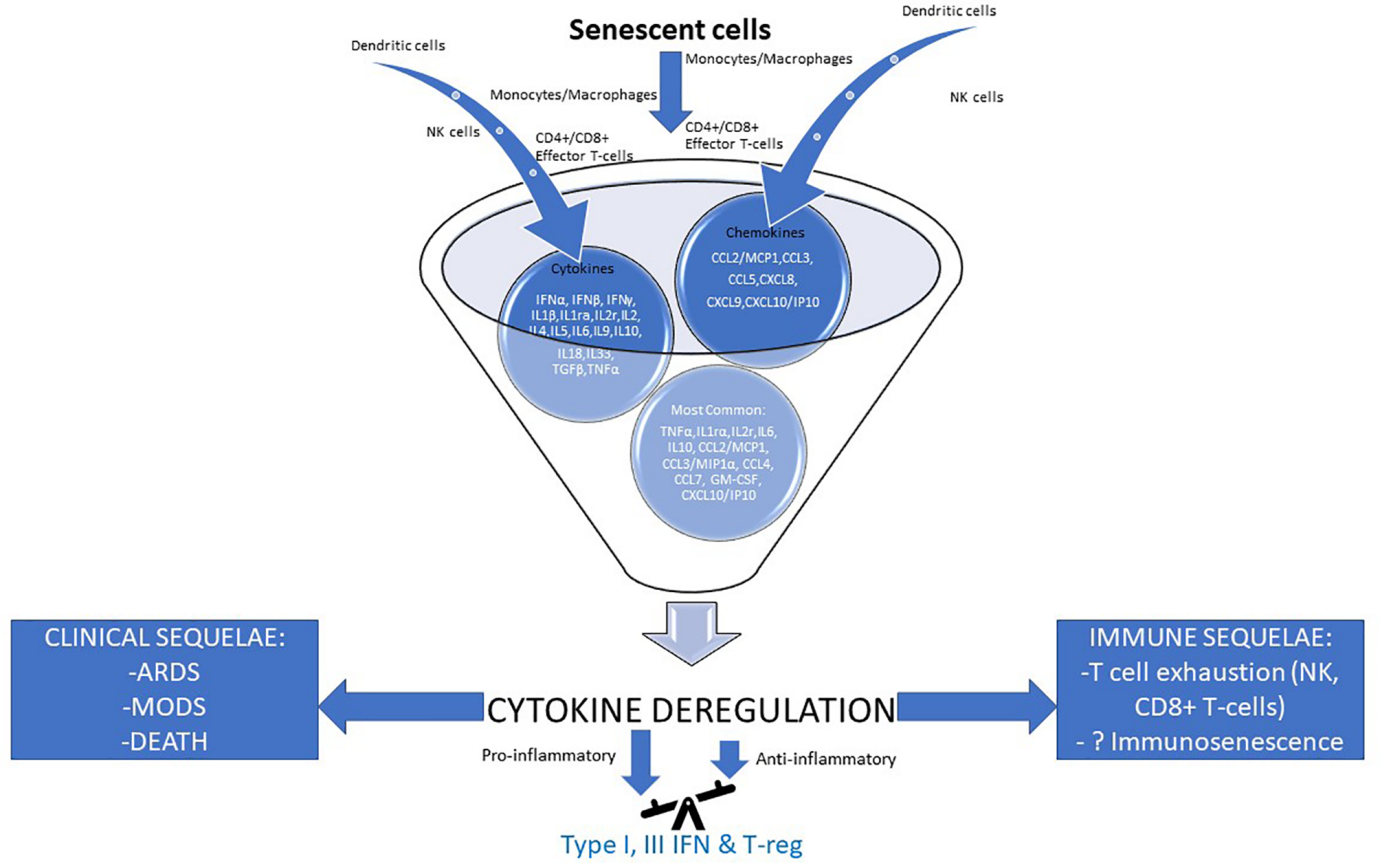
Cytokine deregulation seems to play a pivotal role in the pathogenesis of severe COVID-19. Severe/critical disease outcomes for COVID-19 have been associated with an excessive immune response leading to cytokine deregulation which underpins unfavorable immune (right) and clinical (left) sequelae. Effector cells’ input of cytokines and chemokines is depicted, while their most common profile can also be seen. In the foundation of cytokine deregulation lies an unbalanced pathological immune response with major pro- and little anti-inflammatory contribution secondary to at least in part, impaired IFN type I & III and T-reg actions (87, 110–112). ARDS, acute respiratory distress syndrome; CCL, C–C motif chemokine ligand; CXCL, C-X-C Motif Chemokine Ligand; GM-CSF, Granulocyte-macrophage colony-stimulating factor; IFN, interferon; IL, interleukin; ILr, interleukin receptor; IP10, Interferon gamma-induced protein 10; MCP1, monocyte chemoattractant protein-1; MIP1a, macrophage inflammatory protein alpha; MODS,multiple organ disfunction syndrome; NK cells, natural killer cells; TGF-β, transforming growth factor beta; TNF-a, tumor necrosis factor a; T-reg, regulatory T-cell.

The above-described cytokine deregulation, augmented by the deregulated Renin-Angiotensin-Aldosterone System (RAAS) shown in **Figure 2**, has a series of immunological and physiological effects that eventually lead to multi-organ damage, and if not treated, to multi-organ failure, ARDS and death (**Figure 6**) (87, 110–112).

### Immune Sequelae

#### Deregulated Innate Immunity

Decreased HLA-DR and increased PD-L1 expression has been documented in COVID-19 monocytes (94). These findings suggest exhaustion of these cells and probably impaired antigen presentation to naive T cells (113). DCs in COVID-19 patients are reduced, with impaired maturation and function, suggesting also impaired antigen presentation and T cell activation (114). Patients with severe disease display the highest expression of PD-L1 and the lowest expression of the maturation marker CD80 on DCs (94). Activated NK cells expressing KIRs and CD16 are significantly decreased in COVID-19 patients, implying either impaired maturation or increased migration into the target tissues (115). They also correlate inversely with IL-6 plasma concentrations (116). Moreover, NK cells also show signs of exhaustion, with increased expression of the inhibitory CD94-NKG2A receptor heterodimer and decreased expression of CD107a, IFN-γ, IL-2 and granzyme B (117). Additionally, a low eosinophil count has been correlated to a worse prognosis in COVID-19 patients (118). Finally, increased numbers of myeloid-derived suppressor cells (MDSCs) – pathologically activated monocytes and neutrophils with potent immunosuppressive activity – have been detected in COVID-19 patients, with their number and activity correlating to disease severity (119–122). Such MDSCs have been shown to exhibit enhanced immunosuppressive activity by suppressing T-cell proliferation and function, as well as increased IFN-γ and TNF-a production, probably contributing to a form of immune-paralysis observed in COVID-19 patients (119–121). Interestingly, during the convalescent phase of the disease, the numbers of MDSCs decline, but still remain increased in patients with severe COVID-19 in comparison to patients with mild disease (at least during the initial phase of convalescence) (119).

#### Deregulated Adaptive Immunity

##### T-Cells

CD4^+^ and CD8^+^ lymphopenia is commonly found in COVID-19 patients, resulting possibly either from direct cytotoxic effects of the virus, or from enhanced T-cell apoptosis due to the dysregulated cytokine milieu or metabolic disorders such as lactic acidemia (123).

Decreased T cell counts in severe COVID-19 have been associated with reduced T cell activation and function markers, including TCR subunits (CD3ϵ, CD3γ, CD247, TRAC, TRBC1), surface accessory molecules (CD4, CD8α, CD8β, CD2), T cell migration stimulators (DDP4), TCR signaling kinases (ZAP70, LCK, FYN) and MHC-II molecules (124). Peripheral CD8^+^ T cells also express high levels of exhaustion markers like PD1, TIM3 and CD57, suggesting a functional immune deficiency (125). In severe COVID-19, T cells also show features of an exhausted phenotype. These cells possibly not only lack markers of clonal expansion, but at the same time produce pro-inflammatory molecules (126). Thus, high levels of IL-1a and TNFα promote Th17 responses, increased vascular permeability and leakage. In turn, Th17 cells produce IL-17, GM-CSF, IL-21 and IL-22 (126). IL-17 has broad chemotactic effects through the upregulation of chemokines such as MIP2A, IL-8, IP10 and MIP3A (127).

Surprisingly, a marked plasma increase of typical Th2 cytokines, including IL-4, IL-10, and IL-13 has been observed in some severe COVID-19 patients. This observation could indicate that the activation of immune system in some severe cases, is not Th1-restricted but possess a much broader activation, involving all cells, similarly to what occurs during bacterial sepsis. Such a condition can lead in a form of immuno-paralysis (126, 128).

FoxP3^+^ Tregs are also elevated in several severe COVID-19 patients. It appears that severe COVID-19 entails a striking induction of Treg activation markers including FoxP3, KLRG1 and PD1 and a reduction of CD45RA, a marker of naïve Tregs. Moreover, severe COVID-19 induces the expression of Tbet that preferentially controls Th1 responses and downregulates the inflammatory cytokines TNFα, IL-1β. These changes suggest that Tregs in severe COVID-19 are shaped towards a super-suppressive phenotype equivalent to that observed in tumor Tregs (129).

##### B-Cells

Analyses of circulating B cells in patients with severe COVID-19 have shown an expansion of oligoclonal plasmablasts and reduced frequency of memory B cells (47, 130). The antibody sequences of the largest B cell clones isolated from these patients have variable levels of somatic hyper-mutations and VH gene usage, indicative, as anticipated, of a polyclonal B cell response (130). Many COVID-19 patients possess neutralizing antibodies minimally mutated, suggesting a limited affinity maturation and, consequently, low binding affinity towards SARS-CoV-2 (131). This disparate antibody response might indicate a failure to develop robust long-lasting protective humoral responses against SARS-CoV-2 in some patients (132).

Early antibody responses against the spike protein have been associated with a favorable outcome, while early responses against the viral nucleocapsid with poor clinical outcomes (133). Detailed analysis of the antibody subtypes has revealed elevated IgG1 antibody production with a significantly increased Fc afucosylation. Afucosylated antibodies possess a much higher affinity for the FcγRIIIa receptor, triggering NK cell activation, the production of pro-inflammatory cytokines from monocytes/macrophages, and the cytotoxic effector cell activities provided by cells bearing FcγRIIIa receptor (134). At the same time, afucosylated antibodies against SARS-CoV-2, might contribute to antibody-dependent enhancement of viral infectivity by promoting viral internalization in different cells expressing Fcγ receptor, as it has been shown in animal studies (135).

### Disease Sequelae

#### Endothelial Activation and Endothelitis

The high levels of circulating pro-inflammatory cytokines seen in severe COVID-19 are associated with local and systemic endothelial dysfunction and injury. IL-6 and TNFα can increase vascular permeability and activate the endothelium (24). In turn, endothelial activation leads to increased TF expression, downregulation of thrombomodulin expression and loss of heparin sulfate - all protective mechanisms against thrombosis (136). Direct endothelial cell infection by SARS-CoV-2 and subsequent endothelitis can lead to increased vascular permeability, platelet activation, enhanced thrombin generation and decreased fibrinolysis, contributing to a hypercoagulable state (137, 138). Moreover, SARS-CoV-2 has been shown to directly disrupt the endothelial barrier by altering the surface expression of VCAM1 and the tight junction scaffold proteins ZO-1 and ICAM1, resulting in both chemotaxis and extravasation of inflammatory cells in the perivascular space (139). In COVID-19 patients, the perivascular space around the damaged endothelium is infiltrated by increased numbers of CD3^+^ and CD4^+^ T-lymphocytesand the process of angiogenesis is skewed towards an intussusceptive pattern of vessel formation (140). Moreover, cytokine deregulation can lead to blood-brain-barrier disruption, hence enhancing the entrance of cytokines, or even SARS-CoV-2 into the central nervous system (CNS) (141). This mechanism has been proposed as the main event driving the entrance of the virus into the CNS and the intrathecal production of anti-SARS-CoV-2 antibodies in the cerebrospinal fluid, leading to neurologic damage through either the complement or macrophages (142).

It is hypothesised that the endothelial cell dysfunction, when it occurs in COVID-19, characterises the more severe forms of the disease, underpinning the associated complications including the capillary leak syndrome and thrombosis (143). Such a dysfunction can be caused by endothelial cell inflammation, as shown in autopsy studies (144, 145) which can either reflect direct viral cytotoxic effects or a bystander/indirect effect of hyper-cytokinemia (146). Although the latter is generally well accepted in the literature, direct endothelial cell infection by SARS-CoV-2 remains still elusive.

#### Immunothrombosis

**Figure 7:**
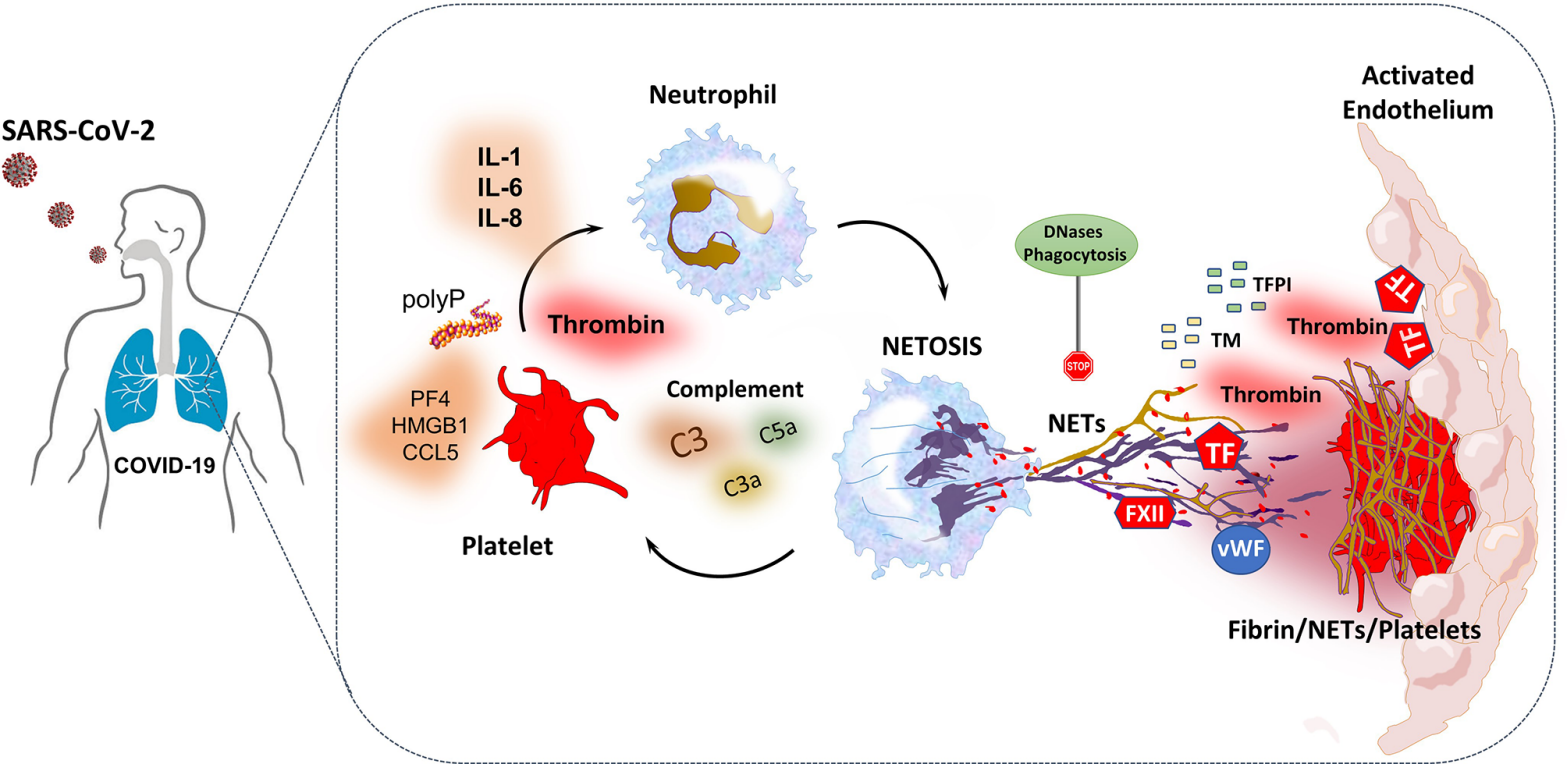
SARS-CoV-2-triggered NETs induce thromboinflammation through interactions with complement system, platelets and the coagulation cascade (161, 163). NETs provide a platform for expression-activation of coagulation factors such as TF and FXII. They also interact with vWF released by endothelial cells and platelets, leading to platelet adhesion and fibrin formation (174). Additionally, NET-derived histones and TF can induce endothelial cell activation and thrombogenicity. Furthermore, NET formation also enhances thrombus stability and lytic resistance, while through the release of neutrophil elastase, degrades TFPI and thrombomodulin and thus dampens anticoagulation (174). Probably, in COVID-19, increased levels of platelet-derived thrombin and factors such as polyphosphate (polyP), high mobility group box 1 (HMGB1), PF4, RANTES, as well as complement and IL-1, IL-6 and IL-8 can trigger NETosis, which in turn activates platelets creating a feedback loop (174, 175). To the contrary, excessive NET formation is homeostatically regulated by anti-inflammatory mechanisms such as NET degradation by DNases, and phagocytic removal by macrophages, thus NET levels may normalize in convalescent patients (163, 174, 176). All the above suggest that the interplay between complement, platelets and NETs may play a central role in the immunothrombosis and ARDS observed in COVID-19 (161, 163, 176). ARDS, acute respiratory distress syndrome; FXII, coagulation factor XII; IL, interleukin; NET, neutrophil extracellular trap; PF4, platelet factor 4; RANTES, Regulated upon Activation, Normal T Cell Expressed and Presumably Secreted; TF, tissue factor; TFPI, tissue factor pathway inhibitor; vWF, von Willebrand factor.

The coagulation system and the immune system are two co-workers, complementing each other to provide host defense and limit the dissemination of invading pathogens (147). COVID-19 is characterized by an elevated risk for thrombosis both in macro- and micro-circulation, contributing substantially to morbidity and mortality (148). Many studies suggest that SARS-CoV-2, through various mechanisms, has the ability to affect different cells or systems in both the immune and the coagulation arm of the host defense, amplifying lung or systemic inflammation and subsequently leading to tissue damage and thrombosis in a process known as immunothrombosis (149, 150). The main contributors to this phenomenon -as depicted in **Table 2** **(** 93, 151–173) and **Figure 7** **(** 149, 161, 163, 174–176) - are: megakaryocyte dyshomeostasis, platelet activation, Neutrophil Extracellular Traps (NETs) formation, complement hyper-activation and coagulation cascade activation as well as endothelial activation/inflammation as described above (149, 150, 163).

**Table 2:** Proposed mechanisms contributing to the (immuno)thrombosis observed in COVID-19 patients.

Effector cells/molecules	Study findings and/or proposed mechanisms	Reference
Megakaryocytes	Increased number and metabolic activity – strong IFN-I expression signature in COVID-19 patients	(151)
	Increased numbers of megakaryocyte nuclei in post-mortem lung sections of COVID-19 patients suggesting *de novo* production of platelets	(152)
	Megakaryocytes are the main drivers of pulmonary neovascularization and fibrosis	Reviewed in (153)
Platelets	Platelets from COVID-19 patients exhibit altered gene expression, increased activation and formation of platelet-leukocyte complexes, and increased release of platelet-derived proinflammatory and prothrombotic microparticles	(93, 154, 155)
	Platelets from COVID-19 patients release larger amounts of proinflammatory cytokines, chemokines and growth factors in comparison to healthy controls upon stimulation	(156)
	Platelets contribute to increased fibrinogen, vWF and FX in COVID-19 patients	
NETs	NET number, as well as their contents, like myeloperoxidase-DNA, and citrullinated histone H3 are significantly increased in COVID-19 patients, correlate with disease severity, and may return to normal in convalescent patients	(157, 158)
	NETs are found in pulmonary microthrombi in patients who died from COVID-19 ARDS	(157, 159, 160)
	NETs provide a platform for expression-activation of coagulation factors such as TF and FXII	(161–163)
	NETs interact with vWF released by endothelial cells and platelets, leading to platelet adhesion and fibrin formation	
	NET-derived histones and TF can induce endothelial cell activation and thrombogenicity	
	NET formation enhances thrombus stability and lytic resistance, while through the release of neutrophil elastase, degrades TFPI and thrombomodulin and thus dampens anticoagulation	
Complement	Uncontrolled activation of the complement pathway may cause endothelial cell injury, thrombosis and intravascular coagulation, leading to a multi-systemic organ failure	Reviewed in (164)
	C4d and C5b-C9 deposits in lung and skin microvasculature are co-localized with viral spike glycoproteins	(165)
	Increased levels of complement system proteins are associated with enhanced activation of neutrophils and subsequent release of NETs and TF	(163)
	The serum levels of C5a and C5b-9 are elevated and correlate to disease severity and vWF levels	(166)
	C3 inhibition downregulates procoagulant and fibrinolytic responses and attenuates NETosis in COVID-19 patients	(167)
	Complement activation correlates strongly with CRP levels, prothrombin time and D-Dimers	Meta-regression analysis in (168)
Coagulation	TF expressed either by the activated endothelium or by neutrophils, macrophages and platelets, can activate the extrinsic coagulation pathway	(149)
	NETs and complement can activate the intrinsic coagulation pathway	(169)
	Complement hyper-activation and several pro-inflammatory or regulatory cytokines, including IL-1β, IL-2, IL-6, IFN-γ and TNF-α promote thrombosis through the endothelial activation, pro-coagulant NETosis, increased release of ultra-large vWF multimers, increased production of FVII/FVIIa, TF and PAI-1, as well as increased generation of thrombin	(170)
	Anti-phospholipid autoantibodies detected in COVID-19 patients are associated with NETosis and have the ability to accelerate venous thrombosis in mice	(171)
	Angiotensin signaling through AT1 can systemically enhance inflammation and thrombosis, and through the activation of the Kinin-Kallikrein system, it can induce systemic fibrinolysis and result in a DIC-like condition	Reviewed in (172)
	Activation of the Kinin-Kallikrein system and the complement cascade are associated with increased D-Dimers and disease severity in COVID-19 patients	(173)

ARDS, Acute respiratory distress syndrome; AT1, angiotensin II receptor 1; DIC, disseminated intravascular coagulation; FVII, coagulation factor VII; FXa, coagulation factor Xa; FXII, coagulation factor XII; IFN-γ, interferon gamma; IFN-I, interferon I; IL, interleukin; NET, Nuclear Extracellular Trap; NETosis, production and release of NETs; PAI-1, plasminogen activator inhibitor 1; PF4, platelet factor 5; RANTES, Regulated upon Activation; Normal T Cell Expressed and Presumably Secreted; TF, tissue factor; TFPI, tissue factor pathway inhibitor; TNF-a, tissue necrosis factor a; vWF, von Willebrand factor.

#### Autoantibodies and Autoimmunity

Some researchers have questioned whether the manifestations of COVID-19 are a mere result of the infection per se or a result of autoimmune reactions initiated or exacerbated by the infection (177). Viral infections not only share common immune responses with autoimmune diseases (ADs), but also they can break immune tolerance and cause autoimmunity by a variety of mechanisms such as molecular mimicry, bystander activation and epitope spreading (178–180). The reduction of regulatory T cellsand the increased number of Th17 and cytotoxic CD8^+^ T-cells that are observed in severe COVID-19 (as described above), have also been described in several autoinflammatory/autoimmune diseases (181, 182). Additionally, lymphopenia and neutrophilia, as well as extrafollicular B-cell activation that have been observed mainly in severe COVID-19, also consist a common feature between COVID-19 and some ADs (183, 184). Moreover, cytokine deregulation and immune hyper-stimulation with some features of immunoparalysis can break tolerance and trigger the activation of auto-reactive T- and B-cells in autoimmune susceptible individuals in an antigen-independent manner, a phenomenon known as bystander activation (185, 186). Additionally, peptide sharing analyses have identified a massive hexapeptide and heptapeptide sharing between SARS-CoV-2 protein S and human proteins, with more than 460 such examples (187, 188). It has therefore been suggested that antibodies against SARS-CoV-2 peptides or against the viral S protein, could boost immune cross-reactions against human proteins like the pulmonary surfactant, the brainstem respiratory pacemaker transglutaminases 2 and 3, mitochondrial proteins, nuclear antigens etc., thus leading to autoimmune tissue destruction (189). Finally, it has been proposed that the enhanced NETosis observed in COVID-19 could also drive autoimmune phenomena, since excessive NETosis has been implicated in the pathophysiology of other autoimmune diseases (186, 190). Interestingly, enhanced expression and circulation of autoantigens like MPO or PRTN3 has also been observed in COVID-19 patients, and the levels of expression of some of these autoantigens correlate with disease severity (191).

In COVID-19 patients, a variety of autoimmune diseases/phenomena have been described, reflecting the overall aberrant immune activation observed in these patients:

1) Different autoantibodies have been detected in many COVID-19 patients with no previous autoimmune rheumatic disease, including antinuclear antibodies, p-ANCA, c-ANCA, anti-CCP, anti-RNP, anti-centromere, Rheumatoid Factors, anti-topoisomerase I and anti-prothrombin, with no, so far, clear clinical significance. Interestingly, some of these antibodies correlate with high levels of CRP and more severe disease. However, whether these antibodies are “functional” and have an etiopathogenic relation to COVID-19 manifestations or are just an epiphenomenon, for the majority of cases, remains to be proven (180, 192, 193).

2) Autoimmune thrombocytopenia or hemolytic anemia with the concurrent detection of warm autoantibodies or cold-agglutinins have also been described, and some of these cases have been successfully treated with IVIG (189). As for the hemolytic anemia, molecular mimicry is probably the main pathogenetic mechanism, since for example, the erythrocyte protein Ankyrin-1 shares a common pentamer with SARS-CoV-2 spike protein (194). Interestingly, IgG3 which is associated with worse prognosis in COVID-19, is the only IgG subclass capable of forming cryoglobulins through Fc-Fc interactions (195). On the other hand, some cases of vaccination against SARS-CoV-2 have been associated with a prothrombotic immune thrombocytopenia (VIPIT) attributed to autoantibodies directed against PF4, a situation similar to Heparin-Induced Thrombocytopenia (HIT) (196).

3) Antiphospholipid syndrome or even catastrophic antiphospholipid syndrome with the involvement of at least 2 target-organs have also been described in COVID-19 patients. Interestingly, not all 3 types of antiphospholipid antibodies (lupus anticoagulant and/or anticardiolipin and/or beta_2_ glycoprotein I) have not been detected in all cases. The levels of antiphospholipid autoantibodies correlated with the CRP levels in serum, D-dimer concentrations in plasma, platelet counts, absolute neutrophil counts, calprotectin in serum (marker of neutrophil activation), and myeloperoxidase–DNA complexes in serum (marker of NETs). Additionally, high levels of such antibodies are associated with more severe disease. However, antiphospholipid antibodies are not always accompanied by thrombosis and in such cases they can possibly be merely the result of the immune hyperactivation and prothrombotic state observed in these patients (171, 192, 197).

4) Some patients have developed different forms of vasculitis (small vessel, middle vessel, large vessel, IgA, cutaneous, CNS vasculitis etc.) in which the inflammation is not limited to the endothelium, but manifests as a peri- and pan-arteritis with immune complex depositions[Reviewed in (186, 198)]. A more severe form of vasculitis is the Kawasaki-like disease (KD-like) or Multisystem Inflammatory Syndrome in children with COVID-19. During the pandemic, a striking rise in KD-like cases has been reported. This disease affects mainly children and targets preferentially the heart, the intestine, and the brain. The pathogenetic cause of this disease seems to be an autoimmune vasculitis, since different autoantibodies have been described in these patients, like antibodies against endoglin, MAP2K2 and members of the Casein Kinase Family, with a distinct inflammatory profile from that of COVID-19 cytokine deregulation (199).

5) Otherpatients have developed an acute inflammatory immune-mediated progressive poly-radiculoneuropathy presenting with tingling, weakness, autonomic dysfunction, and pain, resembling Guillain-Barre syndrome or its variant Miller-Fisher syndrome. Some of these patients have been successfully treated with IVIG or plasmapheresis (189).

6) Cases of inflammatory arthritis or spondyloarthropathy, usually 1-3 weeks after the infection have also been recorded (198).

7) Some COVID-19 patients have developed a new systemic connective tissue disease like Systemic Lupus Erythematosus (SLE). On the other hand, a flare of a preexisting autoimmune disease after COVID-19 has also been described (200–204).

8) Case reports of autoimmune myositis or glomerulonephritis, as well as autoimmune endocrinopathies (thyroiditis, Grave’s disease, type I diabetes mellitus) have also been described in the context of COVID-19 [reviewed in (186, 198)].

9) Some researchers also believe that anosmia (loss of smell) that develops in many COVID-19 patients (186), could also be an autoimmune phenomenon, since such olfactory disturbances have also been described in other autoimmune conditions (205).

10) Based on histopathological, radiological and serological similarities between COVID-19-related ARDS and acute exacerbation of connective tissue disease-induced interstitial lung disease, some researchers suggest that lung involvement in COVID-19 in predisposed patients might be a type of organ-specific autoimmune reaction (206).

COVID-19 in patients already suffering from an AD – either clinically or subclinically – may be affected by this AD or the medications used for it and, to the contrary, may influence this AD. On the other hand, patients without any AD face the danger of developing a new AD as a result of COVID-19 (207). Theoretically, patients with autoimmune diseases are at increased risk for infections and thus increased risk for COVID-19, due to deregulated adaptive immunity (cellular and/or humoral) (208). However, different studies addressing this issue have not clearly established such an increased risk (189, 209, 210). To the contrary, some patients with prior autoimmune/autoinflammatory conditions infected with SARS-CoV-2 have shown better outcomes, probably due to baseline immunomodulatory medications (211–213). For example, the use of TNF inhibitors has been associated with protection from severe COVID-19, while most of the disease-modifying anti-rheumatic drugs (DMARDS) do not seem to confer increased risk (214). On the other hand, patients treated with Rituximab or Secukinumab exhibit high concentrations of IL-6 and seem to have a worse prognosis. CD20 blockers, in particular, could impair B-cell function and therefore the production of neutralizing antibodies against SARS-CoV-2 (215). Chronic sulfasalazine use has also been associated with worse prognosis (216). Moreover, it seems that co-administration of conventional synthetic DMARDSwith biologic treatmentsputs patients at increased risk for severe COVID-19 (217). Finally, chronic treatment with glucocorticoids has been associated with poorer outcomes (218).

Genes associated with genetic susceptibility for autoimmune diseases (like *TLR7*, MHC region, *PTPN22*, *TYK2* and *IL6R*) are being assessed in an effort to explain heterogeneity in COVID-19 severity and organize treatment decisions in a personalized way, but the results are still inconclusive. For example, genetic variations in IL-6 signalling could affect both the presentation of COVID-19 and the response to an anti-IL6 treatment (68).

## Discussion

**Figure 8:**
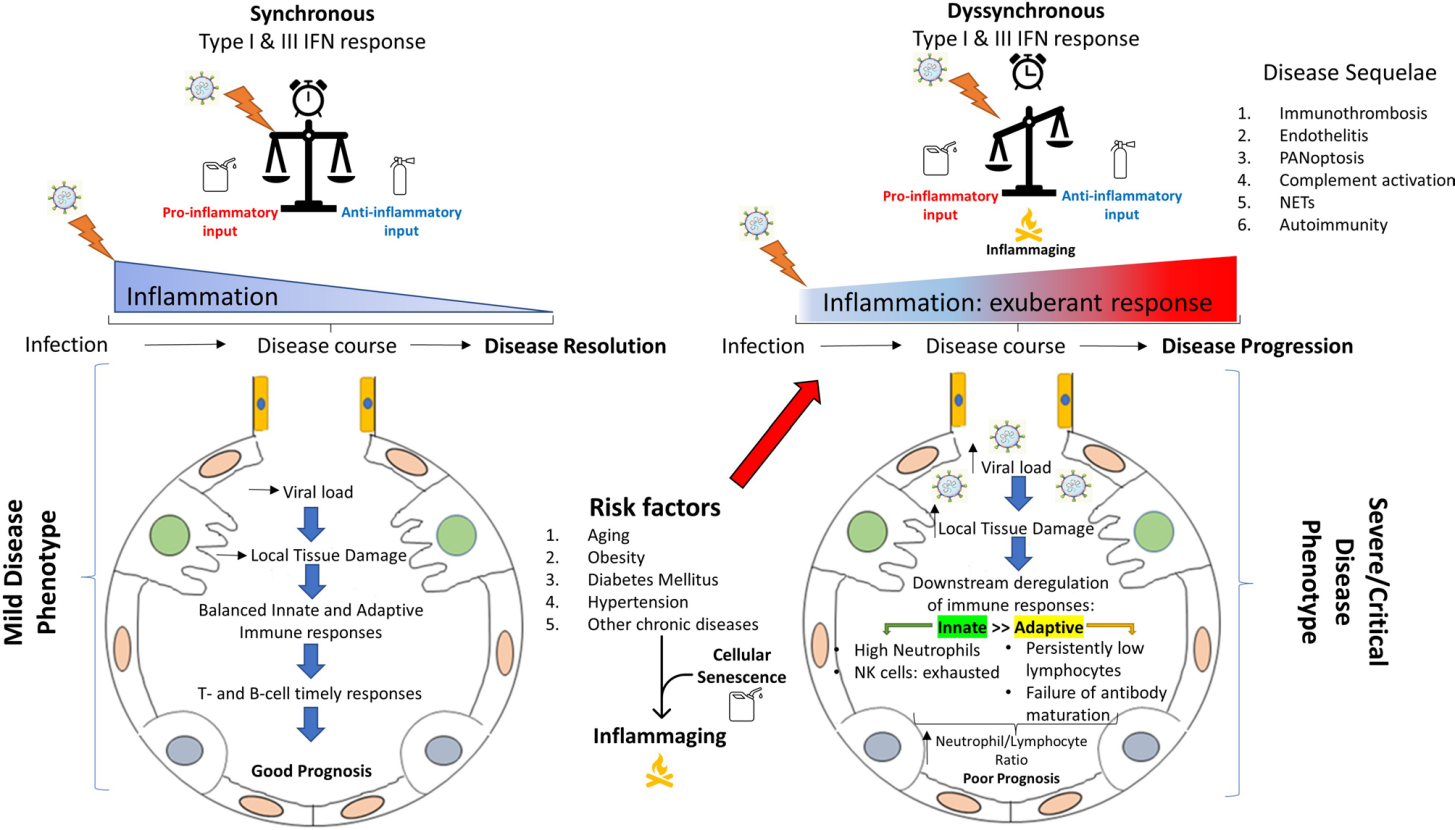
Immunopathological differences between mild *versus* severe COVID-19. A well organized and spatiotemporally synchronized immune response (left), results in viral clearance and disease resolution through collaborative action of innate and adaptive immunity. To the contrary, in severe COVID-19, a series of risk/predisposing factors (like age, obesity, hypertension, diabetes mellitus, cellular senescence etc.) in addition to other still unknown factors, leads to a spatiotemporal deregulation of the immune response (right). Subsequently, this deregulation can result in aberrant and sustained inflammation with multisystemic effects (cytokine deregulation, immunothrombosis, endothelitis, inflammatory cell death, NET production and release, complement activation, autoimmune phenomena etc.), leading to severe disease or even death. NET, neutrophil extracellular trap.

It has been more than 1,5 years now since the initial declaration of COVID-19 as a pandemic. During this time, a great amount of knowledge has been gathered as a result of intensive research from many labs and hospitals around the world. An important message coming out of these studies is that many of the detrimental effects of COVID-19 in end-organ damage/pathology are caused by insults directly attributed to the virus per se, but are also secondary to the immune deregulation (219). In this review, we attempted to summarize in a stepwise approach, the key pathophysiological processes that take place from viral inhalation to disease establishment, with emphasis on the immunopathology of COVID-19 (ranging from immunity to autoimmunity) and its heterogenous clinical outcomes. Intensive ongoing research all over the world, makes the need for continuous update of the acquired knowledge more than essential since new concepts constantly arise (for example PANoptosis or cellular senescence as features of COVID-19 pathobiology). **Figure 8** depicts the critical steps for viral clearance or alternatively for viral persistence and aberrant/sustained immune responses resulting in severe disease and/or death.

Many researchers nowadays suggest, that, the deregulated immune response against SARS-CoV-2, as it is presented in this review, has some characteristic features of autoinflammation and autoimmunity and could therefore be used as a disease-model for this cluster of diseases of unknown etiology (192). A single virus – SARS-CoV-2 – has been shown to possess the capability to drive such a robust hyper-inflammatory immune response that even self-tolerance can be disrupted. As a consequence, and probably through other mechanisms like molecular mimicry, autoantibodies can be produced and result in different autoimmune phenomena and end-organ damage (220). Many researchers believe that the main manifestations of COVID-19 might be attributed to such autoimmune phenomena and not to the infection per se. For example the time needed for the full panel of symptoms to arise (1-2 weeks), coincides with the time needed for the production of autoantibodies (220, 221). Persistence of these autoantibodies and/or the accompanying inflammation after the resolution of the acute phase of COVID-19 has also been proposed as a possible mechanism leading to the so-called “long-COVID” (222–224). On the other hand, it is of interest, that, some of the main drugs used in patients hospitalized with COVID-19 (like corticosteroids, IL6 inhibitors, Jak inhibitors etc.) are immunosuppressive/immunomodulatory drugs commonly used in autoimmune/autoinflammatory diseases (225–229).

Many data that are now available strongly support the concept of COVID-19 being more than an infectious disease, with autoimmunity playing a pivotal role. However, many questions regarding COVID-19 and autoimmunity remain unanswered. On the one hand, complete understanding of the pathogenetic mechanisms underlying COVID-19, will give the opportunity for new, more targeted – thus more effective and less harmful – treatments. For example, blockade of inflammatory cell death (like pyroptosis and PANoptosis) could offer an alternative drug-target for COVID-19 and also help in the treatment of autoinflammatory diseases. Additionally, the temporal distribution of the different immunopathological mechanisms that drive COVID-19 pathogenesis, could help identify the right timing for specific therapies (like interferons). Moreover, if COVID-19 is not dealt with as a purely infectious disease, then other therapies could be implicated in its treatment (like anti-CD20 for the elimination of B-cells, IVIG or plasmapheresis for the neutralization or removal of pathogenic autoantibodies etc.). Detection of susceptibility genes/polymorphisms could also help identify patients at risk for developing severe COVID-19 and autoimmune manifestations and guide treatment in a personalized manner.

On the other hand, it is not known whether all autoantibodies that are created in autantibody-naïve patients have an “active” role, or some of them represent an epiphenomenon. Additionally, neither what happens with these antibodies as time passes after the infection resolution, nor the mechanisms regulating their disappearance or preservation are known. Such knowledge could offer new insights into the treatment of autoimmune diseases. Moreover, since the cases of long-COVID have started to increase, it is essential to understand the pathogenetic mechanisms underlying this condition, to treat it in a more targeted-manner. Such knowledge could probably offer new ideas about the treatment of other chronic conditions like chronic pain or fibromyalgia.

Another promising concept regarding COVID-19 pathobiology and autoimmunity, is the concept of cellular senescence. It has been shown, that cellular senescence, and especially immunosenescence, is associated with the development of autoimmunity (230, 231). Could cellular senescence that preceds COVID-19 (e.g. in the elderly) or is triggered by the disease, put patients at higher risk for developing autoimmunity?

Lymphopenia is regarded as a hallmark of COVID-19 [according to the study and the disease severity, frequency can reach even 85% (232, 233)], with redistribution to sites of active infection and enhanced destruction within the context of cytokinemia being proposed mechanisms. Yet the exact aetiology behind low lymphocyte count is still unknown as well as whether direct SARS-CoV-2 tropism to such cells exists (232). Additionally, studies have demonstrated the presence of spike viral antigen in lymphocytes by immunohistochemistry, while isolation of viral RNA extends beyond lymphocytes to include almost all leukocyte types (234). As a result, leukocytes could be hypothesized to act as a trojan horse facilitating immune evasion and potential viral dissemination systemically. However, appropriate experiments are still lacking to support this notion. In line with that, the functional outcome of direct viral replication within leukocytes would need to be investigated and the fate of those cells to be determined. Could exhaustion of the infected cells take place or immunosenescence be triggered as a response? What is driving immune exhaustion in COVID-19 and what differences exist between mild and severely infected individuals? What is the role of immunosenescence in the establishment of the cytokine deregulation and how does it correlate with clinical outcomes? Does the degree of immunosenescence differ in mild *vs* severe disease, and if so, how?

All the above are some questions that if answered, they would deepen our understanding of the immunological phenomena in COVID-19 and possibly widen our-currently limited-treatment options against it. Scientists are eagerly seeking for new therapeutic agents (235) and drug repurposing tailored to COVID-19 is actively being investigated (236). In the light of findings pinpointing cellular senescence as an important disease modulator, especially in high-risk populations (31, 237, 238), such efforts could be expanded to include experimental use of senolytic drugs as well. This group of medicines act either by apoptosis-induced elimination of senescent cells (senolytics) or inhibiting their SASP-mediated effects (senomorphics) (239). Irrespective of their mode of action, senolytic drugs have tremendous translational potential not only in age-related diseases but in the treatment of infectious diseases too (240). As a stress response triggered by viral infections, senescent cells become tangible targets for elimination with these drugs potentially altering disease course by disrupting viral replication within them and clearing their associated inflammatory properties. Their use, either upon disease establishment or even prophylactically in high-risk populations harbouring a high-burden of these cells, is promising (241). Nonetheless, as these cells are important to normal homeostasis and physiology (99, 101), their destruction comes at a fair cost. To illustrate the latter, the use of senolytics has been associated with reactivation of latent viral infections (242), systemic toxicity (243) and aberrant wound healing (244). Formal studies and clinical trials to assess their clinical efficacy are required prior to their consideration as therapeutic agents. As cellular senescence has a bright and a dark side, depending, at least in part, on the duration of its effects (245), clarifying the specifics of the timing of their interventional use should be a priority.

Concurrently to human studies, a series of pre-clinical animal models (mainly non-human primates, ferrets, Syrian hamsters, and genetically modified mice) have also been used [reviewed in (246–248)]. Unfortunately, these disease-models do not mimic human disease in its entirety. One important difference between humans and animal models of COVID-19, is the fact that most of the animals develop only mild-moderate disease that is limited to the lungs. In most of these cases, the initial response to SARS-CoV-2 consists of a local innate immune activation (macrophages, mononuclear cells, NK cells, neutrophils) in the lungs with the production of inflammatory cytokines like IFN-γ and IL-6 and in some cases with the additional help of residual lymphocytes – especially cytotoxic CD8^+^ T-cells. Moreover, in some of these models, type I and III IFNs have been shown to play a protective role. However, in contrast to humans, no severe cytokine deregulation with robust hyper-cytokinemia (and their consequences) has been described, and the initial phase of immune activation is usually followed by a more anti-inflammatory 2^nd^ phase and disease resolution. These observations can be attributed either to an “inherent weakness” of each animal model (e.g., lack of enough ACE2 receptors for the initial infection to take place at sufficient levels) or to the local immune responses developed in these animals, that manage to eliminate the virus and prevent systemic disease. Interestingly, this milder disease phenotype can be overridden with the use of older animals or use of a higher viral inoculum [reviewed in (249, 250)]. This observation suggests that the immune system has finite “capacity” of dealing with an intruder, and this capacity might decline with age. Further studies in animal models of COVID-19 and interpretation of their results in the light of comparative immunology might give us some new information about the crucial initial immune response that could block SARS-CoV-2 on site without evolving into a severe systemic disease.

## Author Contributions

AK and KB wrote the review. PS, VG, PV and AT substantially contributed to the discussion of the content, reviewed and edited the manuscript before submission. Additionally, KB, PS and PV created the images presented in this manuscript. All authors contributed to the article and approved the submitted version.

## Funding

This work was funded for open access publication fees by the Institute for Autoimmune Systemic and Neurological Diseases, Athens, Greece. VGG and AGT are financially supported by the National Public Investment Program of the Ministry of Development and Investment/General Secretariat for Research and Technology, in the framework of the Flagship Initiative to address SARS-CoV-2 (2020ΣΕ01300001).

## Conflict of Interest

The authors declare that the research was conducted in the absence of any commercial or financial relationships that could be construed as a potential conflict of interest.

## Publisher’s Note

All claims expressed in this article are solely those of the authors and do not necessarily represent those of their affiliated organizations, or those of the publisher, the editors and the reviewers. Any product that may be evaluated in this article, or claim that may be made by its manufacturer, is not guaranteed or endorsed by the publisher.

## Glossary

**Table:**

anti-RNP	Anti-ribonucleoprotein
anti-CCP	Anti-cyclic citrullinated peptide
APOBEC1	Apolipo-protein B mRNA-editing enzyme catalytic polypeptide 1
ARDS	Acute respiratory distress syndrome
c-ANCA	Cytoplasmic antineutrophil cytoplasmic antibody
CCL	Chemokine (C-C motif) ligand
CCR	Chemokine (C-C motif) receptor
CD	Cluster of differentiation
CD3δ	T-cell surface glycoprotein CD3 delta chain
CD3ϵ	T-cell surface glycoprotein CD3 epsilon chain
CD16	Fc fragment of IgG, low affinity receptor III (FcγRIII)
CD45RA	Protein tyrosine phosphatase, receptor type, C (long isoform)
CD57	3-beta-glucuronosyltransferase 1 (B3GAT1)
CD94	Killer cell lectin-like receptor subfamily D, member 1 (KLRD1)
CD107a	Lysosomal-associated membrane protein 1 (LAMP-1)
CD169	sialoadhesin,
CD247	T-cell surface glycoprotein CD3 zeta chain (CD3ζ)
CNS	central nervous system
COPD	chronic obstructive pulmonary disease
CRP	C-reactive protein
CXCL	Chemokine (C-X-C motif) ligand
CXCR	Chemokine (C-X-C motif) receptor
DC-SIGN	Dendritic Cell-Specific Intercellular adhesion molecule-3-Grabbing Non-integrin
DDP4	Dipeptyl peptidase-4 inhibitor,
DNA	Deoxy-ribonucleic acid
dsRNA	Double-stranded ribonucleic acid
ER	Endoplasmic Reticulum
FABP4	fatty-acid binding protein 4
FADD	Fas-associated protein with death domain
Ficolins	Oligomeric lectins with subunits consisting of both fibrinogen (Fi)-like globular domains and collagen (Col)-like long thin stretches with lectin (Lin) activity
FcγRIII	Fc fragment of IgG, low affinity receptor III (CD16)
FoxP3	Forkhead box P3
FVII	Coagulation factor VII
FX	Coagulation factor X
G-CSF	Granulocyte colony-stimulating factor
GM-CSF	Granulocyte/monocyte colony-stimulating factor
GSDME	Gasdermin E
HDAC2	Histone deacetylase 2
HLA	human leukocyte antigen
ICAM1	intracellular adhesion molecule-1
IFIT	*Interferon* Induced proteins with Tetratricopeptide repeats,
IFN	Interferon
IFNAR	Interferon-α/β receptor
IL	Interleukin
iNOs	Inducible nitric oxide synthase
IP-10 (or CXCL10)	Interferon gamma-induced protein 10
IRF	*Interferon regulatory factor*
ISGs	Interferon-stimulated genes
KIRs	Killer-immunoglobulin like receptors
KLRG1	Killer cell lectin-like receptor subfamily G member 1
LAG3	Lymphocyte activation protein 3
LCK	Lymphocyte-specific protein tyrosine kinase
MAC	mMembrane attack complex
MAP2K2	Dual specificity mitogen-activated protein kinase kinase 2
MASP	Mannan-binding lectin serine protease
MBP	Mannose-binding protein
MCP	Monocyte chemoattractant protein
MDA-5	Melanoma differentiation-associated gene 5
MHC	Major histocompatibility complex
MIP-1	Macrophage inflammatory protein 1
MLKL	Mixed lineage kinase domain like
MPO	Myeloperoxidase
MX1	Myxovirus resistance 1
NETs	Neutrophil extracellular traps
NK cells	Natural killer cells
NKG2A-NKG2D	NK group 2 member A-D
NLRP3	NLR family pyrin domain containing 3
NLRs	Nucleotide-binding Oligomerization Domain (NOD)-Like Receptors
NO	Nitric oxide
nsp	Non-structural protein
p-ANCA	Perinuclear antineutrophil cytoplasmic antibody
PAI	Plasminogen activator inhibitor
PAR	Protease activated receptor
PD1	Programmed cell death protein 1
PD-L1	Programmed death ligand 1
PF4	Platelet-factor 4
PRRs	Pattern Recognition Receptors, PRTN3: proteinase 3 (PR3)
RIPK	Receptor-interacting serine/threonine-protein kinase
RLRs	Retinoic acid-Inducible Gene-I-Like Receptors
SPP1	Secreted Phosphoprotein 1 (Osteopontin)
STAT	Signal transducer and activator of transcription
Tbet	T-box *protein* expressed in T cells
TBK1	*TANK-binding kinase 1*
TCR	T-cell receptor
Th	T-helper
TIM3	T cell immunoglobulin mucin-3
TLR	Toll-like receptor
TNFα	Tumor necrosis factor α
TRAC	T cell receptor alpha constant
TRBC	T cell receptor beta constant
TRIM	Tripartite motif-containing
UNC93B1	Unc-93 Homolog B1
VCAM1	Vascular cell adhesion protein-1
Vwf	Von Wilebrand factor
ZAP70	Zeta-chain-associated protein kinase 70
ZO-1	Zonula occludens-1
